# Using EV charging control to provide building load flexibility

**DOI:** 10.1186/s42162-023-00261-8

**Published:** 2023-03-14

**Authors:** Harsimrat Singh Bhundar, Lukasz Golab, Srinivasan Keshav

**Affiliations:** 1 University of Waterloo, Waterloo, Canada; 2 University of Cambridge, Cambridge, UK

**Keywords:** Model-predictive control, Building load shaping, Charging control

## Abstract

Buildings are responsible for a significant fraction of the overall electrical load. Given the increasing penetration of renewables into the generation mix, it is important to make building loads flexible, to better match the variability in generation. Of course, building loads can be made arbitrarily flexible using sufficient stationary storage, but this comes at considerable cost. In this paper, we investigate how to reduce this cost by exploiting electric vehicle (EV) charging control for unidirectional and bidirectional charging. Specifically, we design a model-predictive control algorithm to reshape building load to match a specified load shape. In realistic settings and for two use cases, we investigate the degree to which the amount of stationary storage is reduced using EV charging control. In both cases, we find that our controller reduces the need for stationary storage compared to existing solutions. Moreover, bidirectional EV charging control substantially reduces the required amount of stationary storage.

## Introduction

The weight of public opinion and the rising costs of fossil fuels are placing pressure on grid operators to meet energy needs entirely from renewables (Nehrir et al. 2011). However, renewable generation is inherently variable, so there is a need for loads to be generation-following, i.e., to be flexible.

Buildings are responsible for a significant fraction of the overall electrical load (Sieminski and Administrator 2016). It is therefore critical to make building loads flexible, to better match the variability in generation. Of course, it is possible to make building loads arbitrarily flexible given a sufficient amount of stationary storage, but this comes at considerable cost (Schmidt et al. 2017). To address this problem, we investigate how to reshape a building’s load to match an arbitrary load profile. We do so using a combination of in-building stationary storage and, given the rapid rise in electric vehicle (EV) sales (Hertzke et al. 2018) and the increasing mandate for buildings to provide EV charging facilities in every parking spot, EV charging control (Ardakanian et al. 2014). We focus on buildings with substantial EV penetration, where an EV’s charging load can be reshaped while still allowing it to fully charge by the end of its stay. We also study bidirectional EV charging, given its impending arrival (Sovacool et al. 2017).

Our work differs from prior work on EV charging in two significant ways. First, we are not aware of prior work that focuses on load shape as the control objective; most prior work focuses on objectives such as reducing charging costs or EV peak load. Second, we study buildings with stationary storage and EVs with bidirectional charging, which has not been extensively studied in prior work.

We make the following contributions:

- We design a model-predictive control algorithm that allows a building’s load profile to be reshaped to meet a desired profile by controlling EV charging, taking into account stationary storage and bidirectional EV charging.
- We evaluate the effectiveness of our algorithm in two realistic settings–residential and commercial–using realistic data traces.
- We study the performance of our control algorithm versus existing baselines, with one-way versus two-way charging, with varying amounts of stationary storage and EV penetration levels, and with varying magnitudes of charging duration and building load estimation errors.

The reminder of the paper is laid out as follows. Section "Background and related work" surveys related work. In Sect. "The control algorithm" we present our control algorithm. Section "Experimental design" discusses our experimental setup, followed by the results in Sect. "Results". Finally, Sect. "Discussion and conclusion" concludes with suggestions for future work.

## Background and related work

EV charging places a heavy load on the electrical grid. EVs on the market today can charge at rates ranging from 2 to 150 kW, whereas peak residential electrical load—for example, in North America, consisting of two circuits at 100 A and 110 V—is capped at 22kW, a value that is rarely achieved. Thus, control over EV charging can significantly influence building load shape and flexibility (Pedersen et al. 2018; Schlund et al. 2020). Additional control over load flexibility is possible by using EVs as a source of electrical supply, using Vehicle-to-Grid (V2G) technology (Noel et al. 2019). Thus, in our work, we control both EV and stationary battery charging in a V2G setting to shape the overall building load.

EV charging control has been studied extensively (Solanke et al. 2020; Rahman et al. 2022). However, existing work differs from our work in one of two ways. First, most prior work does not consider load shape control (Solanke et al. 2020; Rahman et al. 2022). As an example, a recent (2020) survey (Solanke et al. 2020) reviews 109 papers on EV charging control, none of which explicitly considers the goal of load shaping. Similarly, the survey in Haupt et al. (2020), which studies charging control for systems that include both EV and stationary storage, does not identify any work on load shape control. Instead, most prior work focuses on coordinating EV charging to reduce negative grid impacts of EV charging, such as transformer overload (Wamburu et al. 2018), grid congestion (Al Zishan et al. 2020; Roy et al. 2021), and load peaks (Mo et al. 2021). Second, only a few papers study systems with both stationary storage and EVs, for example (Haupt et al. 2020), or charging control with V2G (Karfopoulos et al. 2016). However, these papers do not focus on load shape control as the control problem, as we do.

We next focus on the most closely related work (Malhotra et al. 2017; Yao et al. 2013; Anand et al. 2015; Karfopoulos et al. 2016; Haupt et al. 2020). References (Malhotra et al. 2017) and (Yao et al. 2013) focus on coordinated charging of plug-in EVs (PEVs) to shape load in an effort to reduce the negative impacts of uncoordinated charging. However, these works do not consider non-EV loads, bidirectional (V2G) charging, or the presence of stationary (non-EV) storage. Reference (Anand et al. 2015) studies coordinated control of heterogeneous fleet PEVs to reduce peak network loads and takes non-EV building loads into account. It finds that coordinated charging using a hierarchical control implementation meets substation constraints while offering an up to 64% reduction of energy cost to consumers. However, this work does not exploit stationary storage or bidirectional charging, as we do. Reference (Karfopoulos et al. 2016) proposes a distributed EV charging coordination mechanism to support system operation by tracking day-ahead schedules. It finds that coordination of distributed charging can efficiently meet the EV aggregator’s day-ahead scheduling profile using both an adaptive droop-based approach and a proposed distributed regulation dispatch algorithm. It does take V2G into account, but not stationary storage. Moreover, the optimization goal is cost minimization rather than load shape control.

In terms of our control algorithm, the closest work is the Adaptive Charging Network (ACN) (Lee et al. 2021), which focuses on controlling EV charging to maximize site operator revenue and end-user comfort. The authors find that their scheduling algorithm can improve operator profit by 3.4 times over uncontrolled charging in realistic settings. Their system also outperforms baseline algorithms in highly congested systems. However, the control objective is different from ours. Another closely related work is Reference (Haupt et al. 2020), which manages both EV and stationary storage charging, but for cost reduction. They find that for their test environment of an EV charging facility in Augsburg, Germany, the size of stationary storage is a critical parameter. A large amount of stationary storage is necessary if more than 65% of the EVs begin to charge immediately but controlled charging can drastically reduce the required storage size. This related work gives us the confidence that we might expect similar gains from our work.

To sum up, we are not aware of prior work that studies the design of a building load control system that exploits both bidirectional EV charging as well as stationary in-building storage to match a desired load shape. We fill this research gap by proposing an algorithm that allows a building’s load profile to be reshaped to any desired generation profile by controlling EV and stationary storage charging.

## The control algorithm

Our model-predictive control (MPC) algorithm builds on the scheduling algorithm proposed for the Adaptive Charging Network (ACN) (Lee et al. 2021), and adds bi-directional EV charging and stationary storage. We present the details below, with the notation summarized in Table 1.

**Table 1:** Notation

Symbol	Meaning
$\delta$	Duration of a time step
*k*	Time index
${\mathcal {L}}$	Duration of optimization horizon
*l*	Index into optimization horizon
*i*	Index into the set of EVs
$SoC^{\max }$	Capacity of the stationary storage
*SoC*(*k*)	State of charge of stationary storage at time step *k*
$L^{G}(k)$	Desired power profile at time step *k*
$L^{BL}(k)$	Non-EV building load at time step *k*
$\hat{e}_i(k)$	Estimated remaining energy demand at time step *k*
$\hat{d}_i(k)$	Estimated remaining duration of stay at time step *k*
$L^*$	Target load curve for the day
$r_i(k)$	Charging rate of EV *i* at time step *k*
$r^{SS}$	Charging rate of stationary storage
$\bar{r}$	EV maximum charging rate
$\bar{r}^{SS}$	Maximum charging rate of stationary storage
${\mathcal {V}}_k$	Set of actively charging vehicles at time step *k*
$SoC_i(l)$	State of charge of vehicle *i* at time step *l*
$SoC_i^{max}$	Maximum capacity for vehicle *i*
*M*	Number of days in the testing period

We divide a day into $\delta =$ 5-minute time steps. Time is indexed by $k \in {\mathcal {K}} = \{0, 1, 2,$ $\dots , K-1$}. At each time step, the MPC computes a charging rate trajectory over a optimization horizon indexed by *l*, $l \in {\mathcal {L}} = \{0, \dots , L-1\}$; *l* is relative to the current time *k*.

### Assumptions

We make two assumptions in our work. First, we assume that for each day, the MPC algorithm is given the forecast for the non-EV building load profile for the day, $L^{BL}(k)$. This can be obtained using one of many known building load forecasting algorithms (Nti et al. 2020; Zhang et al. 2021). In Sect. "Impact of building load forecast errors", we examine the impact of forecasting errors on the performance of our solution.

On arrival of an EV indexed by *i*, the control algorithm is informed of the EV’s energy demand $\hat{e}_i(k)$ using the ISO 15118 protocol (Multin 2018). Our second assumption is that at arrival time, an EV’s estimated remaining duration of stay $\hat{d}_i(k)$is known. In Sect. "Sensitivity analysis of estimated EV duration", we study the impact of prediction errors in EV duration of stay on performance.

### Algorithm

At each time step *k*, the output of the MPC consists of (1) the charging or discharging rate for the stationary storage, denoted $r^{SS}(k)$, and (2) the EV charging or discharging rates, denoted $r_i(k)$, for each EV *i* in ${\mathcal {V}}_k$, the set of vehicles currently present. These rates are computed such that the net building load (EV + non-EV) for that time step best matches the desired power profile for the day.

Algorithm 1 shows the pseudocode. *SCH* (line 3) is the optimizer that computes an optimal charging schedule for EVs and stationary storage for the optimization horizon (see below). Line 5 updates the estimated remaining energy demand by subtracting the energy delivered over the time step, $r_i(k)\delta$. We lower-bound this by 0 in case the estimate was too low. Line 6 similarly updates the estimated duration of stay.
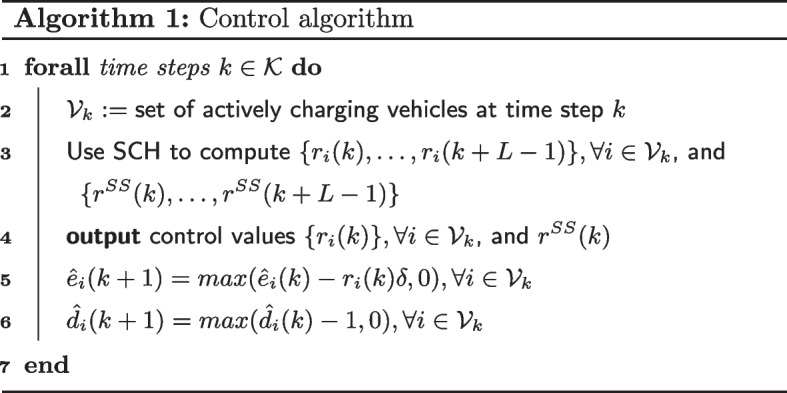


### SCH

We now describe SCH, the online optimization problem. The target load curve for the day, denoted $L^*$, scales the desired load shape for that day ($L^G$) by the non-EV building load for the day ($L^{BL}$), so that this load is met, but has the desired shape. Note that we do not scale by the EV load since this is unknowable in advance. Thus,

$$\begin{aligned} L^*(d) = \frac{\sum _{d \in {\mathcal {D}}} L^{BL}(d)}{\sum _{d \in {\mathcal {D}}} L^{G}(d)} L^{G}(d) \end{aligned}$$

for $d \in {\mathcal {D}}$, where ${\mathcal {D}} = \{1, \dots , \frac{1440}{\delta }\}$ indexes the 5-min time steps of the given day *D*. The optimization objective is to minimize, at each time step *l* over the control horizon, the squared L2-norm of the error between the desired load $L^{*}(l)$ and the actual load $L^{BL}(l) + \sum _{i}r_i(l) + r^{SS}(l)$:

$$\begin{aligned} u^{LM}:= ||L^{*}(l) - (L^{BL}(l) + \sum _{i \in {\mathcal {V}}_k} r_i(l) + r^{SS}(l))||_{2}^2 \end{aligned}$$

This optimization is subject to the following constraints:

1 $$\begin{aligned}{} & {} 0 \le r_i(l) \le \bar{r}, l \in {\mathcal {L}} \end{aligned}$$

2 $$\begin{aligned}{} & {} \sum _{l \in {\mathcal {L}}} r_i(l)\delta \le \hat{e}_i(k) \end{aligned}$$

3 $$\begin{aligned}{} & {} 0 \le SoC(l) \le SoC^{max}, l \in {\mathcal {L}} \end{aligned}$$

4 $$\begin{aligned}{} & {} {}-\bar{r}^{SS} \le r^{SS}(l) \le \bar{r}^{SS}, l \in {\mathcal {L}} \end{aligned}$$

5 $$\begin{aligned}{} & {} r^{SS}(l) \delta = SoC(l) - SoC(l-1), l \in {\mathcal {L}} \end{aligned}$$

1. The charging rate for each vehicle *i* at time step *l* is within the maximum rate, $\bar{r}$.
2. The total energy supplied to vehicle *i* over the control horizon is less than or equal to its estimated remaining energy demand when charging starts at time step *k*. The inequality implies that a vehicle may not be fully charged if this allows us to better match the desired load shape. We explore the impact of this decision, compared to an equality constraint, in Sect. "Impact of fully charging EVs".
3. The state-of-charge of the stationary storage is within 0 and its maximum capacity, $SoC^{max}$.
4. The charging rate for the stationary storage is within its minimum/maximum charging rate, $\bar{r}^{SS}$.
5. The charging rate for the stationary storage at time *l* is the state-of-charge at time *l* minus the state-of-charge at time $l - 1$.

These constraints only consider uni-directional charging. For bidirectional charging, we replace constraints (1) and (2) with the following:

6 $$\begin{aligned}&-\bar{r} \le r_i(l) \le \bar{r}, l \in {\mathcal {L}} \end{aligned}$$

7 $$\begin{aligned}&0 \le SoC_i(l) \le SoC_i^{max}, l \in {\mathcal {L}} \end{aligned}$$

8 $$\begin{aligned}&r_i(l)\delta = SoC_i(l) - SoC_i(l), l \in {\mathcal {L}} \end{aligned}$$

Here, $SoC_i(l)$ is the state of charge of vehicle *i* at time *l* and $SoC_i^{max}$ is the maximum capacity for vehicle *i*.

We study two types of bidirectional charging, with and without ‘borrowing’. Consider the discharge of energy from the EV subsequent to a prior charging. If the amount of energy discharge exceeds the amount of energy charged, we dub this ‘borrowing’. In our experimental results, we study the effect of borrowing on the performance of the system.

## Experimental design

This section presents the data sources, assumptions and metric we used to evaluate our control algorithm. Our goal is to be realistic in the choice of data sources and to model both residential and commercial settings.

### Non-EV building load traces

For residential building load, we use the Apartment Dataset from the University of Massachusetts (2017), which contains power consumption for 114 apartments from 2014 to 2016. We sum the consumption across the 114 apartments to obtain a building load that mimics a typical condominium building. We selected 30 days of data from this dataset that had a 5-minute data granularity. With the combined charging and load data, we simulate a multi-unit residential building, with one chargepoint per parking spot.

For commercial building load, we use 30 days of hourly consumption data for an office building in Toronto, Canada, collected between February 1 2020 and March 1 2020 by our industry partner, SWTCH. The office building was built in 1981. There are 206 parking spaces and 9 floors, for a total gross area of 254,000 square feet. To hyper-resolve the consumption data from hourly to 5 min, we use cubic spline interpolation (Allik and Annuk 2017).

The residential and commercial load shapes are shown in Fig. 4a and b respectively (blue curves). These daily load shapes (building, desired and ours) are averaged over the 30 days of the testing period of our experiments.

### Charging session traces

We did not have access to charging session data from the same location as the building load traces. Instead, we use a trace of residential charging sessions from Electric Chargepoint Analysis 2017, with 3.2 million charging sessions from 25,000 chargepoints (Electric Chargepoint Analysis 2017). These traces are from various locations in the UK. The data consist of arrival time, energy demand, duration and the chargepoint ID for each charging session. The chargepoint ID serves as a user ID in a residential setting and allows us to separate charging sessions by specific users. We eliminated outliers below the 5%-ile and above the 95%-ile values, corresponding to likely errors in the data: very short sessions lasting a few minutes or very long sessions lasting days. To match the 114 apartments in the building load dataset, we use data from 114 chargepoints with at least 40 charging sessions over a 90-day period between January 31, 2017 to March 1, 2017.

For commercial charging sessions, we use 30 days of data, from December 3, 2018 to January 1, 2019, selected from the ACN dataset (Lee et al. 2019) for the JPL site in Pasadena, California, a workplace. The data consist of arrival time, energy demand, departure time and the user ID. We use arrival time and departure time to calculate duration.

### Charging rates

In the residential setting, the maximum charging/discharging rate for EVs is set to $\bar{r} = 7.4$KW, which is common for level-2 residential chargers. The time step is $\delta = 5$ minutes and the optimization horizon is 12 h; that is, ${\mathcal {L}} = \{1, 2, \dots , 144\}$. The maximum charging rate for stationary storage is $\bar{r}^{SS} = 300$KW, which is reasonable for modern buffer units. In the commercial setting, the maximum charging rate for EVs is $\bar{r} = 22$KW, which is common for office charging.

### Metrics

We want to measure the degree to which the controlled load shape matches the desired load shape. We therefore define our accuracy metric ${\mathcal {M}}$ (denoted %match in the results) as:

$$\begin{aligned} {\mathcal {M}} = 1 - \frac{1}{M} \sum _{i=1}^{M} \frac{1}{|{\mathcal {D}}|} \sum _{d} \frac{|L_i(d) - L_i^{S}(d)|}{L_i(d)} \end{aligned}$$

where *M* is the number of days in the testing period (i.e., 30 days), $d \in {\mathcal {D}}$ where ${\mathcal {D}} = \{1, \dots , \frac{24 \cdot 60}{\delta }\}$ indexes time steps of the given day,

and

$$\begin{aligned} L^S = \frac{\sum _{d \in {\mathcal {D}}} L^{BL}(d) + \sum _{i}r_i(d) + r^{SS}(d) }{\sum _{d \in {\mathcal {D}}} L^{G}(d)} L^{G}(d) \end{aligned}$$

${\mathcal {M}}$ subtracts from one the average (over *M* days) of the mean absolute percentage deviation each day between the building’s actual load *L* and renewable supply load scaled to the building’s total (EV+ non-EV) consumption $L^S$ (computed ex post). This gives a measure of similarity of a building’s daily load shape to the desired load shape.

To compute the degree to which a typical day might deviate from the mean computed above, we calculate the standard deviation of the statistic over the *M* days, and the corresponding 95% confidence interval. In the figures that follow in Sect. "Results", shaded areas represent this confidence interval.

Finally, our results plot the metric ${\mathcal {M}}$ on the Y axis with respect to the amount of stationary storage provided on the X axis. Thus, a higher curve is better, in that it achieves a better metric with less stationary storage. All result curves asymptote to 100%, because with a sufficient amount of stationary storage, a perfect goodness metric can always be achieved.

### Default choices

We assume a residential setting by default, except in Sect. "Comparison of residential and commercial settings". We assume uni-directional EV charging by default, except in Sect. "Impact of bidirectional charging".

### MPC implementation

We implemented the MPC using custom software written in Python. The optimization problem was solved using the Gurobi solver.

### Desired load shapes

We use two desired load curves to evaluate our approach. The first is a constant load set to the mean value of the next day’s load, similar to the control goal in Reference (Wang et al. 2022). The second is a renewable load profile derived from the California Independent System Operator (CAISO) renewable supply generation curve, from January 31 to March 1, 2019, scaled as discussed in Sect. "The control algorithm". The load profile was retrieved from http://www.caiso.com/todaysoutlook/pages/supply.aspx. This load profile is illustrated in Fig. 4a and b (orange curve). The renewables are primarily solar and wind, with smaller contributions from geothermal, biomass, and hydro. Note that we focus on renewable energy from the grid rather than from self-consumption.

## Results

This section presents our experimental results. We start by computing the amount of stationary storage needed to meet a desired load shape in the absence of EV charging control (Sect. "Stationary storage needed without EV charging control"). We then compare our EV charging solution to an optimal solution with full knowledge of the future (Sect. "Comparison of our solution to offline optimal"). Next, we compare the behaviour of our algorithm in residential and commercial settings (Sect. "Comparison of residential and commercial settings"), and for unidirectional and bidirectional charging (Sect. "Impact of bidirectional charging"). Section "Comparison to experimental baselines" presents a comparison of our solution to experimental baselines. We then analyze the sensitivity of our approach to EV penetration (Sect. "Performance under different levels of EV penetration"), summer vs. winter months (Sect. "Comparing summer and winter months"), building load forecast errors (Sect. "Impact of building load forecast errors"), and errors in the estimated duration of EVs (Sect. "Sensitivity analysis of estimated EV duration"). We conclude with an impact analysis of enforcing the EV energy demand-supply inequality, i.e., Equation 2 from Sect. "The control algorithm" (Sect. "Impact of fully charging EVs").

### Stationary storage needed without EV charging control

To begin, we study the benefit of using EV charging control in reducing the amount of stationary storage required to meet a desired load shape. We assume that when there is no EV charging control, EVs would charge at the maximum rate ($\bar{r}$) when they arrived and stop when they were full.

**Fig. 1:**
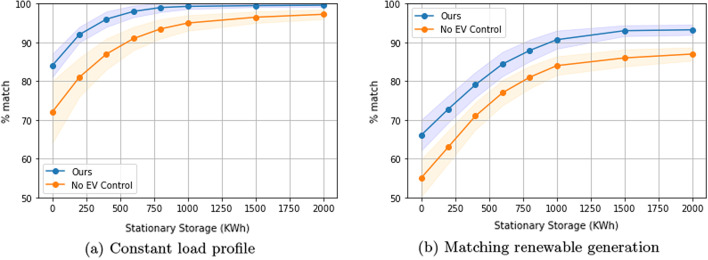
Comparison of our solution to no EV charging control over different levels of stationary storage. One-way charging in a residential setting

Figure 1a and b show that there is a statistically significant improvement in performance using our algorithm to when there is no EV charging control (residential setting, one-way charging, constant load profile). Without EV charging control, the need for stationary storage increases substantially.

As an example, we see in Fig. 1a that in the residential setting with one-way charging (constant load profile), to achieve above a 90% match in desired load shape, our solution only requires 200KWh of stationary storage compared to the 600KWh of stationary storage required when there is no EV charging control.

### Comparison of our solution to offline optimal

**Fig. 2:**
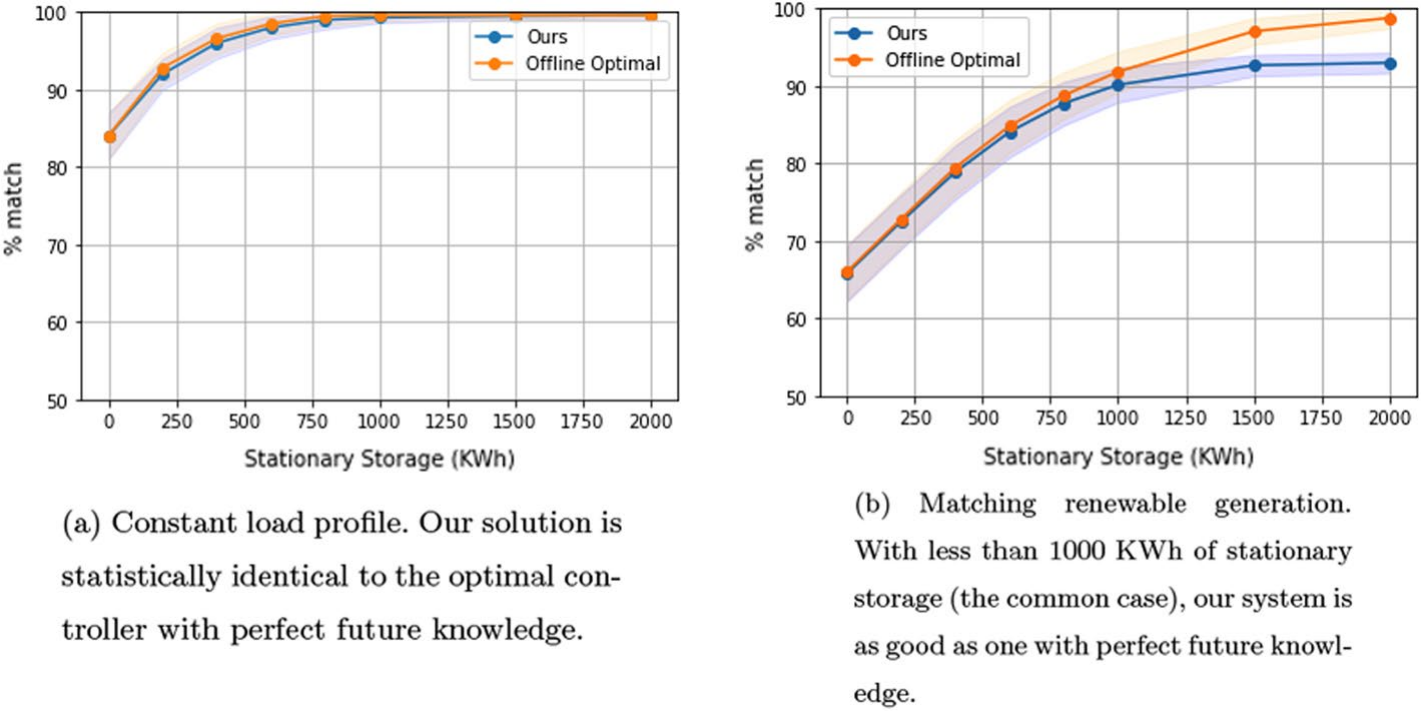
Comparison of our solution to offline optimal. One-way charging in a residential setting

We now compare our algorithm’s performance with an offline optimal schedule computed with perfect future knowledge of EV energy demand and charging duration (Fig. 2a, b).

When the goal is to achieve a constant load shape (Fig. 2a), our solution is statistically indistinguishable from the offline optimal.

Figure 2b shows the results when trying to match the renewable generation curve. With 1000 KWh or less of stationary storage (which is likely to be the case in practice), our solution matches the performance of the offline optimal algorithm that has perfect knowledge of the future. If very large batteries are available, our solution deviates only about 8% from the ideal load shape, whereas the optimal solution is able to perfectly achieve the desired load shape.

### Comparison of residential and commercial settings

**Fig. 3:**
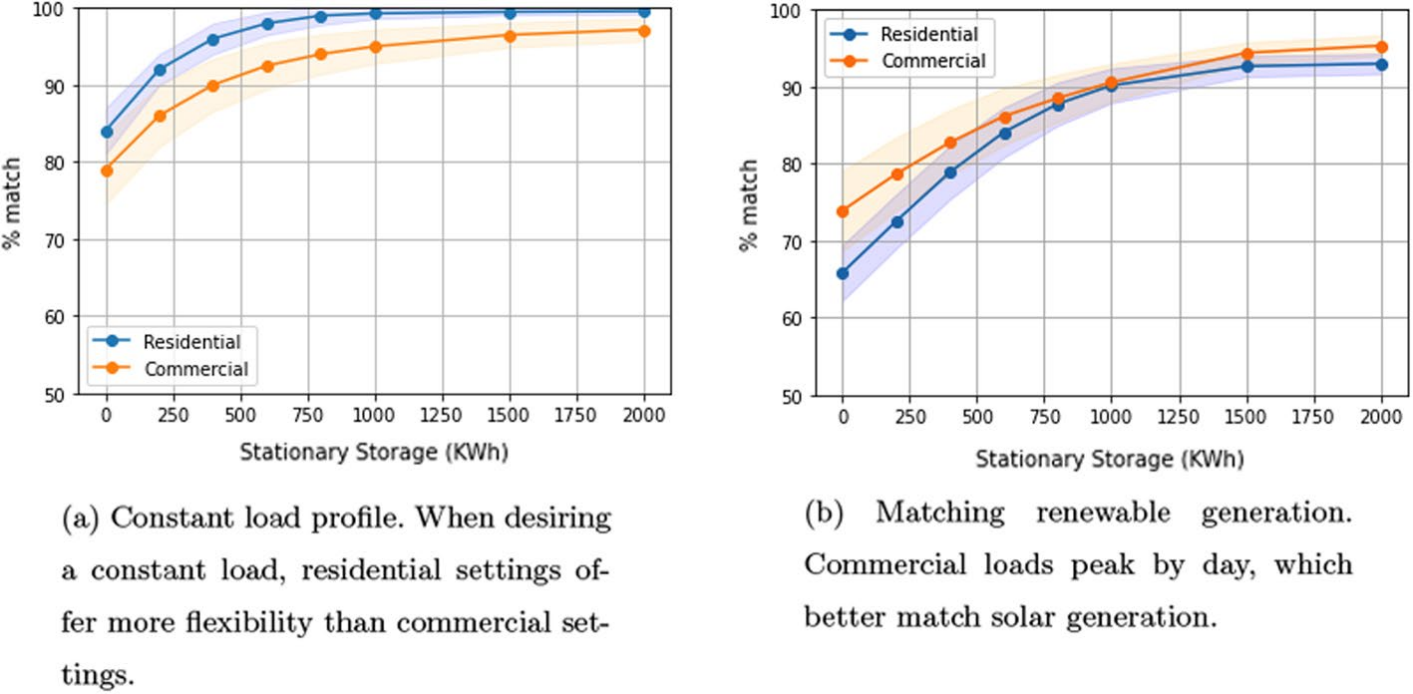
Commercial vs residential settings

We next study differences in our algorithm’s performance in shaping a building’s load curve to match the renewable supply curve in a residential setting compared to a commercial setting. Figure 3a and b compare the performance of our system as a function of stationary storage size in both residential and commercial settings.

Note that, for a constant desired load, performance in a residential setting is better than in a commercial setting, but for maximizing renewable supply, the commercial setting shows better performance. The difference in performance has to do with the desired load shape. When trying to achieve a constant load, deferring of charging over extend time periods of 8–12 h in a residential setting can be exploited to smooth the overall load. In commercial settings, there is less time (the working day) to re-order charging times.

**Fig. 4:**
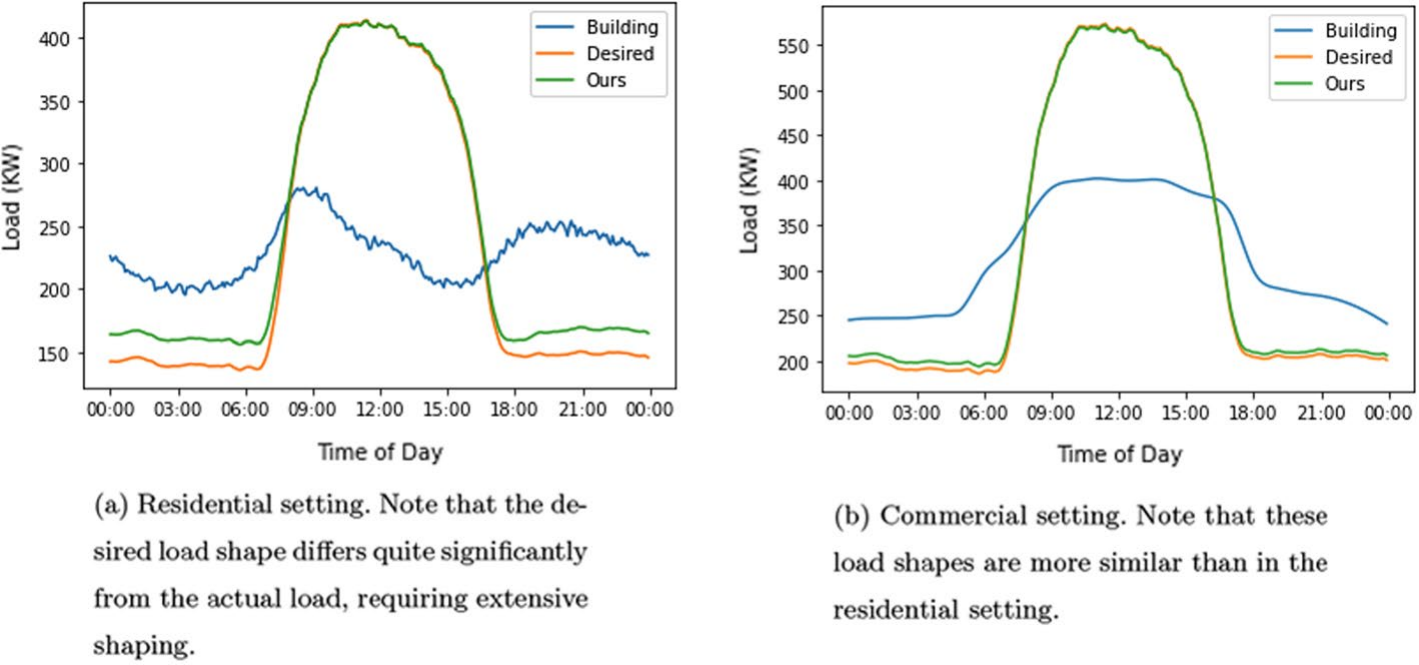
Comparison of average actual load and desired load shapes (matching renewable generation)

In contrast, when trying to maximize renewable (solar) energy use, with low levels of stationary storage, the performance in the commercial setting exceeds the residential setting due to the similarity in the commercial building’s average load shape with the renewable supply load. Specifically, Fig. 4a and b show the building average load shapes (blue), the desired load shapes, which correspond to average renewable shapes (orange), and the obtained load shapes (green) using 750KWh of stationary storage in the residential and commercial settings. Note that the commercial building load shape (Fig. 4b) is closer to the desired load shape than the residential building load shape (Fig. 4a).

### Impact of bidirectional charging

**Fig. 5:**
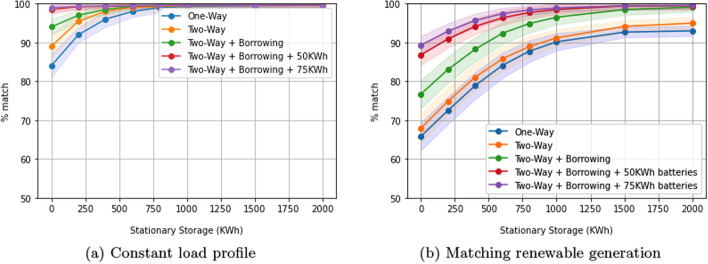
Impact of bidirectional charging in a residential setting. Bidirectional charging helps if ‘borrowing’ is allowed

We compare unidirectional vs. bidirectional charging control in a residential setting in Fig. 5a and b. Both figures show the deviation from the desired load shape as a function of stationary storage size for:

- Uni-directional charging (blue)
- Bidirectional charging, assuming that we never draw more energy from an EV than we supply to it (orange)
- Bidirectional charging assuming we can borrow energy from an EV as long as its SoC always exceeds both the initial SoC and 50%. For this, we introduce the constraint $SoC_i(l) \ge \min (SoC_i(m), 0.5 \cdot SoC_i^{max})$, where *m* is arrival time. We assume that the capacity of each EV is 25KWh, which is representative of existing EVs (green)
- As in (c), but assuming that the capacity of each EV is 50KWh (red) or 75KWh (purple), which represents newer EVs.

For a constant desired load shape (Fig. 5a), we find that with bidirectional charging alone there is little significant change in flexibility if there is no borrowing. However, there is a significant improvement in flexibility when energy is borrowed. This is because we can borrow energy to supplement an immediate shortage between actual and target load. This indicates that allowing an EV to ‘borrow’ stored energy has merit.

As with the constant load shape, for the case of trying to match the renewable load (Fig. 5b), when no energy is borrowed from EVs, there is no statistically significant difference in performance between bidirectional and uni-directional charging. This is because of the necessarily limited amount of charging in EVs with small ( 25 kWh) batteries. With larger storage levels, bidirectional charging leads to significant performance increases at each level of stationary storage, with the performance becoming nearly ideal as the amount of stationary storage increases.

**Fig. 6:**
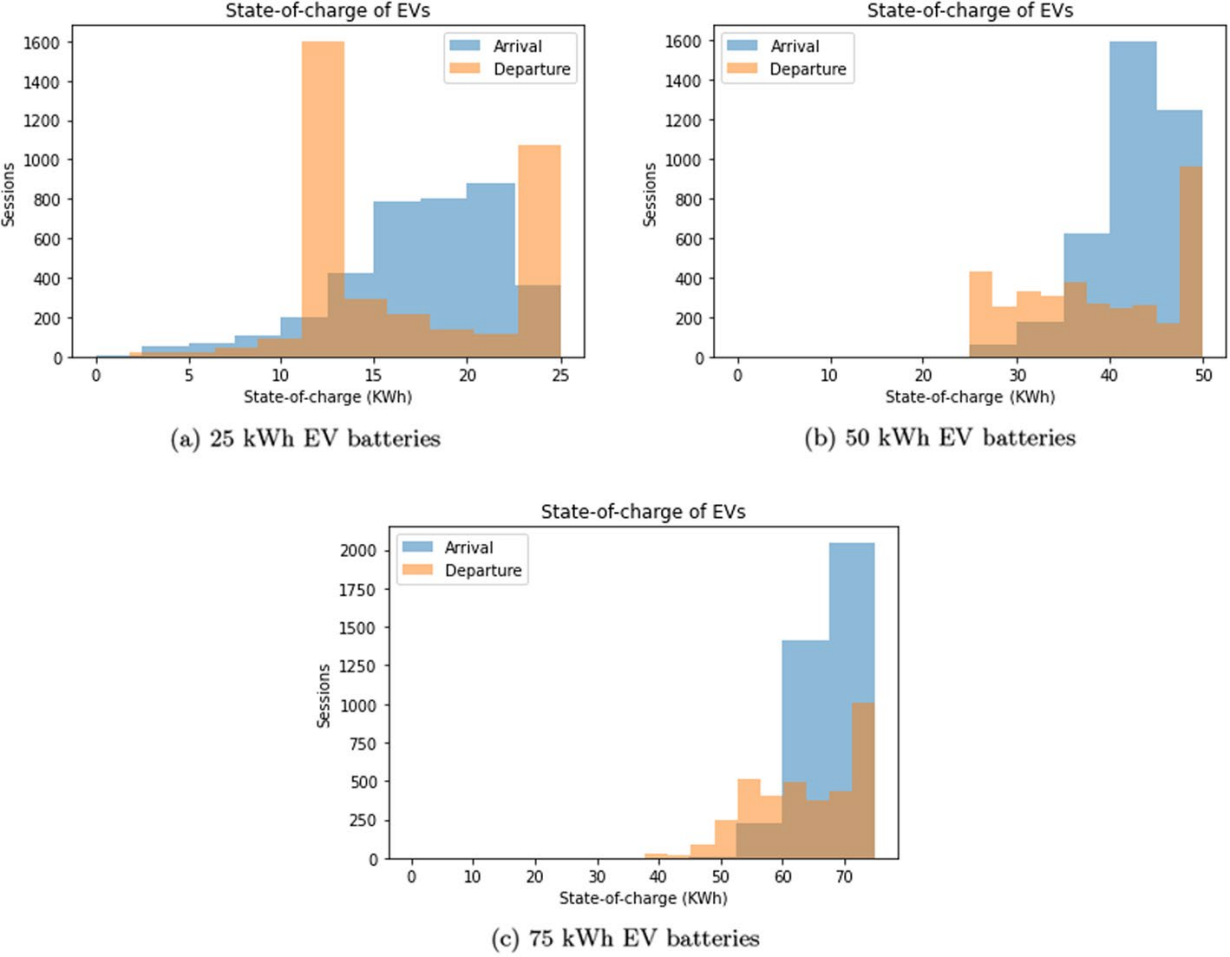
State-of-charge distributions at time of arrival and departure. Two-way charging with ‘borrowing’ in a residential setting (matching renewable generation)

Note that bidirectional charging with borrowing leads to performance increases but may impact end users, in that it could reduce the level of available charge at the time of EV departure. We now show the state-of-charge distribution for the EVs upon arrival (blue) and departure (orange), for EV capacity of 25KWh (Fig. 6a), 50KWh (Fig. 6b) and 75KWh (Fig. 6c). In all cases, we assume there is no stationary storage, so the system can only utilize EV batteries for storage, an extreme situation. In all figures, EVs arrive close to being fully charged, whereas the distribution of state-of-charge on departure is bimodal: most sessions end with the EV being halfway or fully charged, with the latter being more common for larger batteries (Fig. 6b, c). This indicates that borrowing does have a user impact for smaller battery sizes, which represents a tension between user satisfaction and system performance. However, this tension is reduced with larger battery sizes, a situation that is rapidly becoming more common in recent years.

### Comparison to experimental baselines

In this section, we compare the performance of our algorithm against two baselines.

In the case of the constant load profile, we compare our algorithm against the algorithm used in ACN (Lee et al. 2021). We fix the price of the load, and set it to the mid-peak price used in ACN, which is $0.092/kWh. We selected this value for consistency with the experimental setup of ACN. The intuition is that since price is fixed, and ACN’s objective function incentives simultaneous peak reduction and cost minimization, the resulting load curve should become flat as we increase stationary storage.

In the case of matching renewable energy, we also compare our algorithm against the algorithm used in ACN (Lee et al. 2021), only this time the price of the load is set dynamically based on the distance of the actual load curve to the desired load curve. We do this by setting the price at time *t*, denoted *p*(*t*), to the absolute percentage distance between the desired load and the actual load multiplied by the mid-peak price used in ACN, which is $\hat{p} = 0.092$. We get $p(t) = \hat{p} \frac{|L^G(t) - (L^{BL}(t) + \sum _{i \in {\mathcal {V}}_k}r_i(t) + r^{ss}(t))|}{\sum _{t} L^G(t)}$. Note that the denominator is the sum of the desired load for that day, or the total desired consumption. Thus, at any given time, the difference in load is represented as a proportion of the consumption. We scale the dynamic price function with the mid-peak price, $\hat{p} = 0.092$. Again, we selected this value for consistency with the experimental setup of ACN. The intuition for this pricing mechanism is to assign a cost to deviating from the desired load. Since the objective function incentivizes cost minimization, the resulting load should track the desired load profile as we increase stationary storage.

**Fig. 7:**
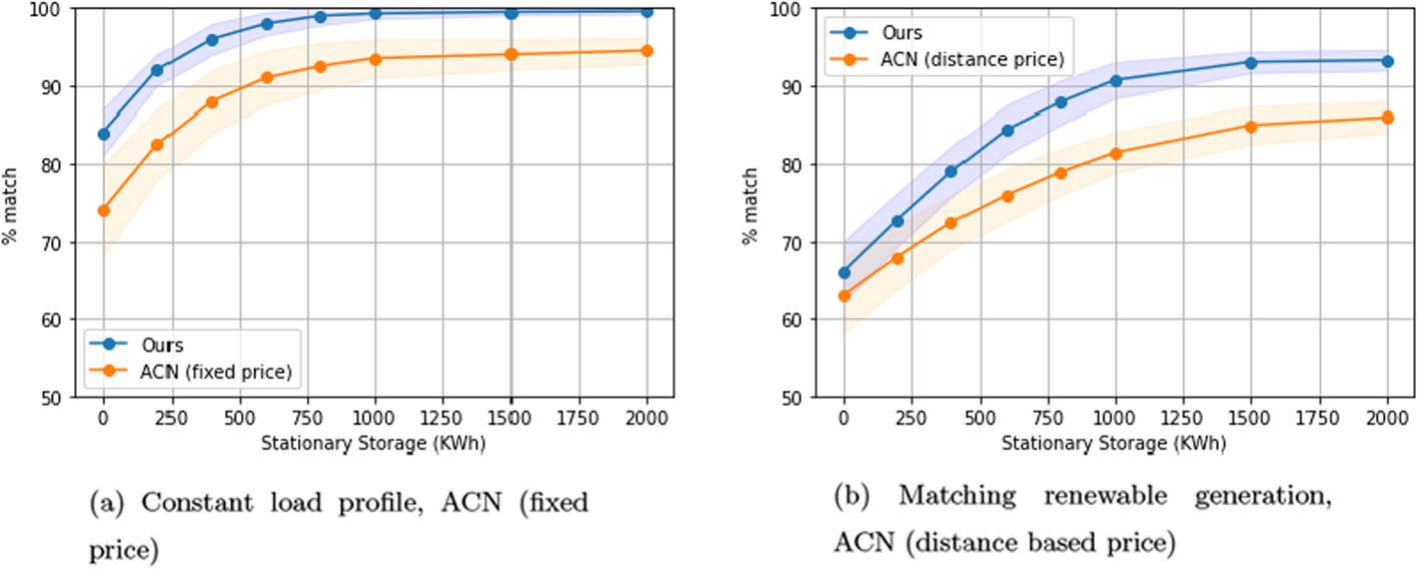
Comparison of our solution to ACN over different levels of stationary storage. One-way charging in a residential setting

In the case of constant load, our solution outperforms the experimental baseline across all levels of stationary storage (Fig. 7a). This can be attributed to competing secondary objectives in the ACN objective function, which includes delivering energy to the charging network as quickly as possible. In the case of matching renewable energy supply, our solution outperforms ACN for stationary storage levels beyond 400KWh (Fig. 7b). Under the dynamic distance based pricing mechanism, cost can be minimized by either reducing the distance between the desired load and the actual load, or by reducing the actual load itself. This results in the controller fluctuating between tracking the desired load and reducing the actual load, leading to an unstable penalty, which is suboptimal when tracking the desired load is the primary goal.

### Performance under different levels of EV penetration

**Fig. 8:**
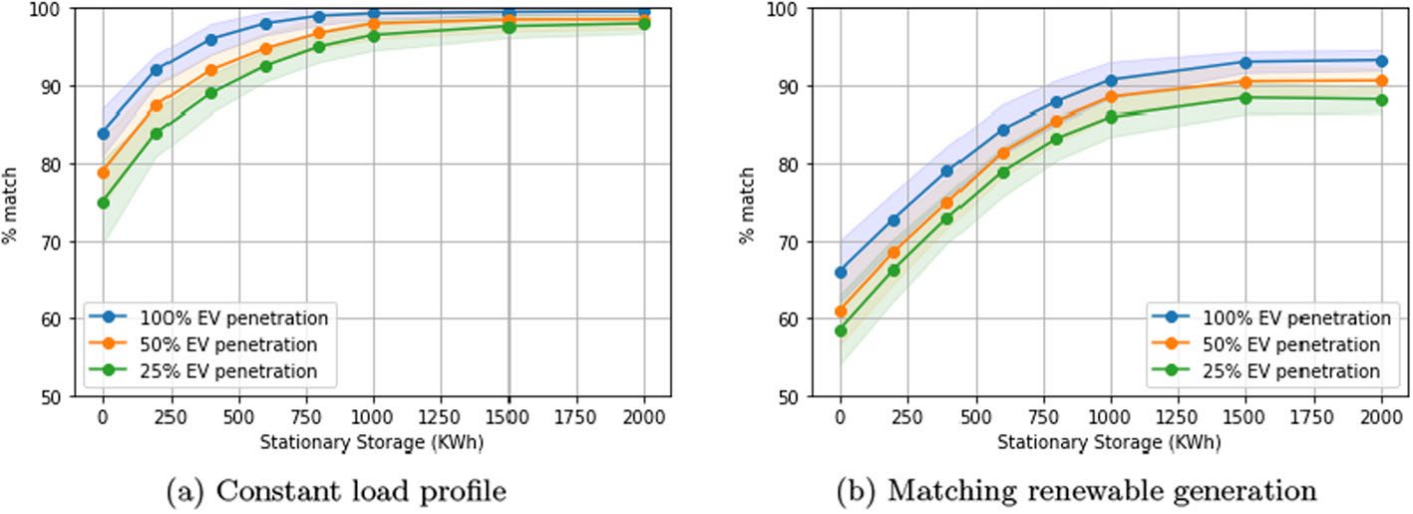
Performance under lower levels of EV penetration over different levels of stationary storage. One-way charging in a residential setting

We now study the performance of our solution under smaller levels of EV penetration. We do this by reducing the sample of EVs for both the commercial and residential building to 25% and 50%. We see there are significant gaps in performance when EV penetration is at 25%, for levels of stationary storage below a 1000KWh in the case of the constant load profile (Fig. 8a). This gap closes as stationary storage increases. In the case of matching renewable generation, there is a significant gap across all levels of stationary storage (Fig. 8b).

### Comparing summer and winter months

**Fig. 9:**
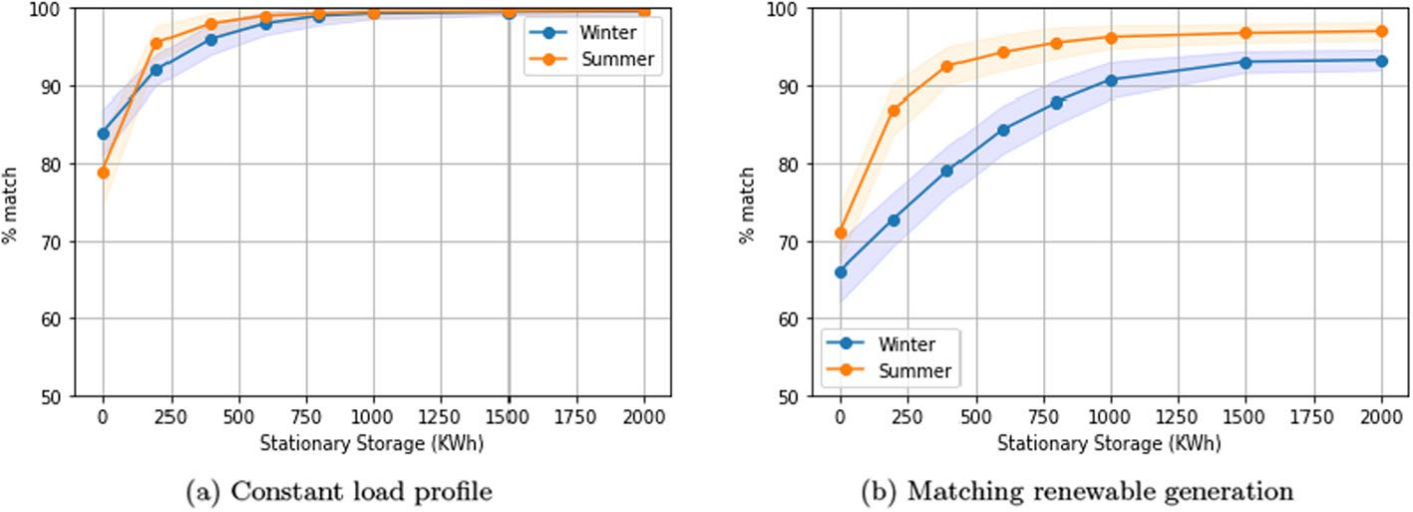
Comparison of the performance of our solution in winter and summer months. One-way charging in a residential setting

The testing period for our default setting across all experiments considers 30 days of winter. In this section, we include results from testing during 30 days of summer (from the month of July). For the residential building, the consumption decreases significantly during summer months. Specifically, during the 30 day summer testing period, the average daily load peak is roughly a third of what it is in the 30-day winter testing period (the default setting). As shown in Fig. 9a, there are no significant changes in performance between summer and winter in the case of the constant load profile. However, in the case of matching renewable generation, there is a significant performance increase in the summer (Fig. 9b). This can be attributed to the decrease in building consumption, which reduces the extent to which load reshaping is required in absolute terms, allowing the system to reach high levels of performance at lower levels of stationary storage.

### Impact of building load forecast errors

**Fig. 10:**
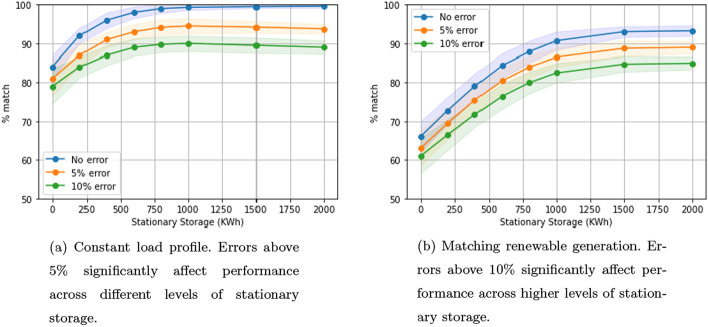
Impact of errors in estimating building load. One-way charging in a residential setting

We study the impact of errors in forecasting building load. Our algorithm receives inputs for the future building load in advance. In a realistic setting, these inputs are from forecasts that may suffer from errors. We study our algorithm’s robustness to these errors by considering two scenarios: errors of 5% and 10%. In both scenarios, at any given time the input for future building load is randomly and uniformly overestimated or underestimated by p% (either 5 or 10). We study this under two settings: constant load profile and matching renewable energy. In both cases, performance deviates significantly for higher levels of stationary storage, especially in the case of the constant load profile (Fig. 10a, b). This is because the system’s ability to meet the erroneous objective improves with higher resources, that is, at higher levels of stationary storage.

### Sensitivity analysis of estimated EV duration

**Fig. 11:**
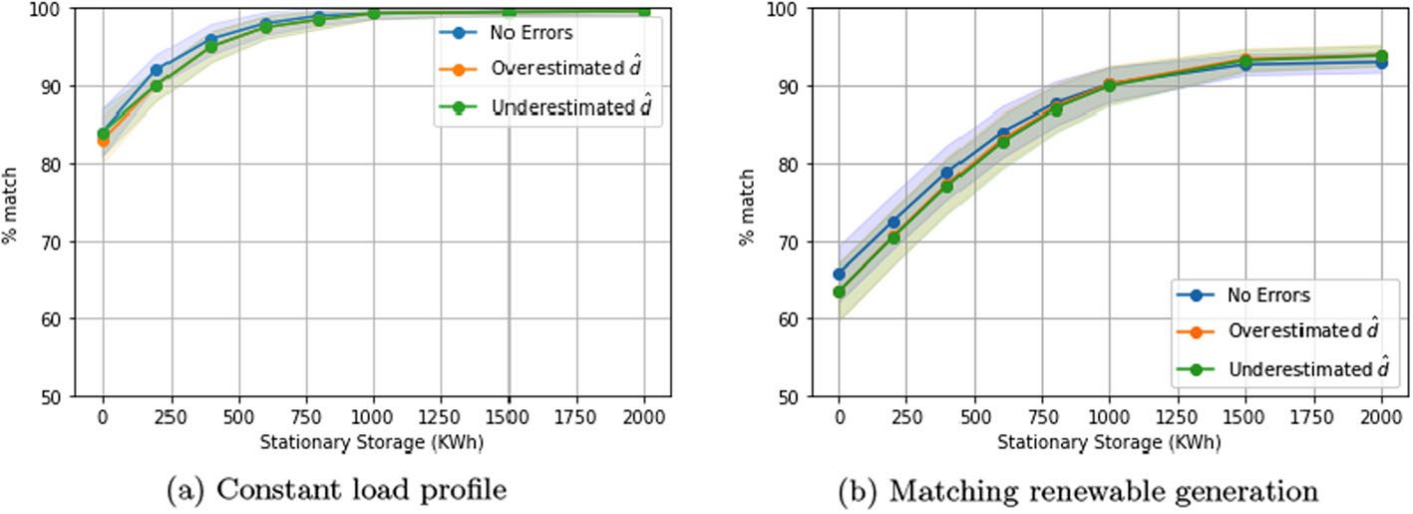
Impact of errors in estimating duration. Errors of up to 25% in charging duration estimation do not significantly affect performance. One-way charging in a residential setting

We now study how errors in estimating duration impact our algorithm’s performance. As mentioned in Sect. "The control algorithm", we receive inputs for estimated EV duration from end users, which may be imprecise. We test the robustness of our algorithm to these errors by considering two scenarios: overestimating and underestimating duration of charge for each EV by 25%, which is a high level of absolute error, since most EVs in a residential setting charge for long durations and often overnight. Figure 11a and b illustrate the impact of duration errors on performance as a function of the stationary storage size. We find that there is no significant difference, showing robustness against errors as high as 25%. This is due to the quadratic penalty term in the optimization objective, which evenly distributes charging across the EVs.

### Impact of fully charging EVs

Note that in Equation (2), reproduced below, we do not guarantee that the energy supplied to an EV is the energy it demands. Specifically, when summed over the control horizon ${\mathcal {L}}$, the energy supplied to EV *i* in time step *l*, i.e., $r_i(l) \delta$, is only upper bounded by the remaining energy needed by the vehicle $\hat{e}_i(k)$:

$$\begin{aligned} \sum _{l \in {\mathcal {L}}} r_i(l)\delta \le \hat{e}_i(k) \end{aligned}$$

To ensure that energy supplied to EV *i* matches its remaining energy demand $\hat{e}_i(k)$ or, if this is infeasible, the vehicle is supplied with the maximum possible energy that can be supplied during its remaining duration of charge $\hat{d}_i(k)$, we replace Equation (2) with:

9 $$\begin{aligned} \sum _{l \in {\mathcal {L}}} r_i(l)\delta = \min (\hat{e}_i(k), \hat{d}_i(k) \cdot \bar{r} \delta ) \end{aligned}$$

where $\bar{r}$ is the fastest possible charging rate.

**Fig. 12:**
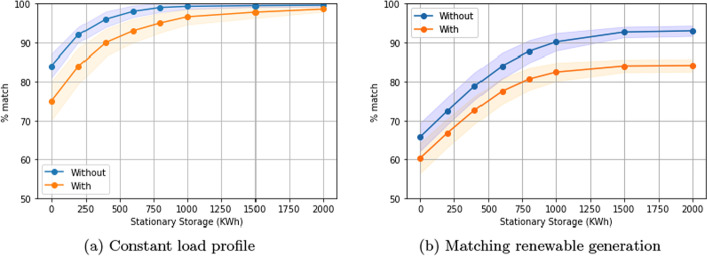
Performance with and without fully charging EVs. The equality constraint reduces performance by at most 10%. One-way charging in a residential setting

When this constraint in introduced, so that EVs are fully charged when feasible, both scenarios show a decrease in the performance metric (Fig. 12a, b). This is because, in this situation, the need to charge EVs trumps the need to meet a target load shape. Nevertheless, even with this change, the performance metric decreases by only about 10% at most for the entire range of stationary storage values tested.

## Discussion and conclusions

Buildings are already significant consumers of grid energy, and, with the introduction of electric vehicles, their demand on the electrical grid is set to further increase. However, by using stationary storage and controlling EV charging, building loads can be made flexible, so that they can better match irregular renewable supply. Our insight is that control over EV charging and bidirectional EV charging can reduce the need for costly stationary storage.

We presented a model-predictive control algorithm to reshape building load to match either a constant or variable load. Experimental results show that our method can reduce the need for stationary storage and that bidirectional EV charging, especially with ‘borrowing’ and larger EV storage, can achieve excellent load shaping even with relatively small amounts of stationary storage (Fig. 5a, b). Our solution performs as well as an algorithm that has perfect knowledge of the future and is insensitive to errors of up to 25% in estimating the duration of charge.

Our controller works best for commercial buildings whose load shapes are more similar to the renewable generation supply curves used in our experiments. Although our results are preliminary, we believe that they are encouraging, and suggest that, with suitable controllers, building loads can be made quite flexible, so that they become supply following, reducing the carbon intensity of the grid. How to put this flexibility to use is an open question for future work.

Our work is not without limitations. First, we study a specific set of load and generation traces. It is possible that performance metrics may differ as functions of these traces. Second, our approach makes a trade-off between the level of EV charging and load shape: if EVs need to be maximally full, then the load shape deviates by at most 10% from the desired goal. We believe this is acceptable. Third, we have evaluated our results using building load data and EV charging data that are not from the same location. Finally, we have not conducted a cost-benefit analysis of our system. We hope to address these limitations in future work.

## Data Availability

In this paper, every dataset is publicly available with the exception of one – office building load – which is proprietary. Section 4 includes citations to all the publicly available datasets. Source code is available from the corresponding author on reasonable request.
